# Population-based cohort study of oral contraceptive use and risk of depression

**DOI:** 10.1017/S2045796023000525

**Published:** 2023-06-12

**Authors:** T. Johansson, S. Vinther Larsen, M. Bui, W. E. Ek, T. Karlsson, Å. Johansson

**Affiliations:** 1 Department of Immunology, Genetics and Pathology, Science for Life Laboratory, Uppsala University, Uppsala, Sweden; 2 Centre for Women’s Mental Health during the Reproductive Lifespan – Womher, Uppsala University, Uppsala, Sweden; 3 Neurobiology Research Unit, Copenhagen University Hospital, Rigshospitalet, Denmark; 4 Department of Clinical Medicine, University of Copenhagen, Copenhagen, Denmark; 5 Centre for Epidemiology and Biostatistics, Melbourne School of Population and Global Health, University of Melbourne, Melbourne, VIC, Australia

**Keywords:** depression, epidemiology, mental health, women

## Abstract

**Aim:**

Research on the effect of oral contraceptive (OC) use on the risk of depression shows inconsistent findings, especially in adult OC users. One possible reason for this inconsistency is the omission of women who discontinue OCs due to adverse mood effects, leading to healthy user bias. To address this issue, we aim to estimate the risk of depression that is associated with the initiation of OCs as well as the effect of OC use on lifetime risk of depression.

**Methods:**

This is a population-based cohort study based on data from 264,557 women from the UK Biobank. Incidence of depression was addressed via interviews, inpatient hospital or primary care data. The hazard ratio (HR) between OC use and incident depression was estimated by multivariable Cox regression with OC use as a time-varying exposure. To validate causality, we examined familial confounding in 7,354 sibling pairs.

**Results:**

We observed that the first 2 years of OC use were associated with a higher rate of depression compared to never users (HR = 1.71, 95% confidence interval [CI]: 1.55–1.88). Although the risk was not as pronounced beyond the first 2 years, ever OC use was still associated with an increased lifetime risk of depression (HR = 1.05, 95% CI: 1.01–1.09). Previous OC use were associated with a higher rate of depression compared to never users, with adolescent OC users driving the increased hazard (HR = 1.18, 95% CI: 1.12–1.25). No significant association were observed among adult OC users who had previously used OCs (HR = 1.00, 95% CI: 0.95–1.04). Notably, the sibling analysis provided further evidence for a causal effect of OC use on the risk of depression.

**Conclusions:**

Our findings suggest that the use of OCs, particularly during the first 2 years, increases the risk of depression. Additionally, OC use during adolescence might increase the risk of depression later in life. Our results are consistent with a causal relationship between OC use and depression, as supported by the sibling analysis. This study highlights the importance of considering the healthy user bias as well as family-level confounding in studies of OC use and mental health outcomes. Physicians and patients should be aware of this potential risk when considering OCs, and individualized risk–benefit assessments should be conducted.

## Introduction

An estimated 151 million women of reproductive age use oral hormonal contraceptives (Haakenstad *et al.*, 2022), of which many women benefit in terms of avoiding abortions and unwanted pregnancies, as well as menstrual bleeding and pain disturbance (David and Kling, 2020). Clinical evidence indicates that hormonal contraception can affect some women’s mood (Payne, 2003), yet the link between using hormonal contraception and depression remains inadequately addressed (Robakis *et al.*, 2019; Schaffir *et al.*, 2016). Several studies have identified an association between hormonal contraception use during adolescence and an increased risk of depression (Anderl *et al.*, 2022, 2020; de Wit *et al.*, 2020; Skovlund *et al.*, 2016; Zettermark *et al.*, 2018). The effects of hormonal contraceptive use on depression risk in adults are less clear, with some suggesting either no increased risk (Cheslack-Postava *et al.*, 2015; Duke *et al.*, 2007; Lundin *et al.*, 2022) or a decreased risk of depression (Keyes *et al.*, 2013; Toffol *et al.*, 2012).

Within the last decade, large-scale population-based studies have explored the association between hormonal contraceptive use and depression. A Danish study on more than one million women found a higher risk of depression for all types of hormonal contraception across all age groups, with the largest risk among adolescents (Skovlund *et al.*, 2016). Similarly, a Swedish study on 800,000 women found a positive association between hormonal contraceptive use and the use of any type of psychotropic drug but only among adolescents (Zettermark *et al.*, 2018). A Swedish study conducted on 900,000 women discovered that different types of hormonal contraceptives were linked to increased use of antidepressants among adolescents, but in adults, this association was only observed for progestin-only compounds. Moreover, the use of combined contraceptives appeared to have a protective effect and was associated with a lower risk of depression (Lindberg *et al.*, 2012). Similarly, a more recent study based on 740,000 Swedish women found that combined oral contraceptives (OCs) were associated with a lower risk of depression when current OC users were compared to nonusers (never and previous users). However, when never users were used as the reference group, this association was no longer significant (Lundin *et al.*, 2022).

In contrast to the observational studies, randomized clinical trials have shown little or no effect of hormonal contraception on mood (de Wit *et al.*, 2021). However, most of these studies did not consider the previous use of hormonal contraception. As highlighted in several studies (de Wit *et al.*, 2021; Skovlund *et al.*, 2016; Zettermark *et al.*, 2018), one limitation in most previous studies is the potential influence of a healthy user bias. Mood effects of OCs can lead to discontinuations and is a contraindication for their use (Larsson *et al.*, 1997), which may result in decreased participation of affected women in subsequent clinical trials and underestimation of effects. Similar underestimation of effects can be seen in observational studies not considering previous OC use, as exemplified in the Netherlands Study of Depression and Anxiety cohort, which found that current OC use in a between-person analysis was associated with lower risk of depression, while the within-analysis showed that the time during OC use was associated with an increased risk of depression. This discrepancy is likely explained by a healthy user bias as those negatively affected by OC use had discontinued and thus represented nonusers in the between-person analysis (Morssinkhof *et al.*, 2021). A study that specifically focused on long-term effects observed that adolescent OC users had a higher prevalence of depression several years after initial exposure, compared to never and adult OC users. These findings indicate that adolescence could be a susceptible phase for OC use to heighten the lifetime depression risk (Anderl *et al.*, 2020).

Inconsistent findings in previous studies may be explained by healthy user bias. Research that accounts for this type of bias by, for example, considering the temporality between the first initiation of OCs and depression incidence is needed. Using a “new user” design approach (Yoshida *et al.*, 2015), as opposed to a “prevalent user” approach that includes both current and new users, avoids underestimating the effect of exposure. The present study was therefore designed to estimate both the incidence rate of depression associated with the first initiation of OC use but also the lifetime risk that is associated with OC use.

In this study, we utilize medical information from more than 250,000 UK Biobank (UKB) women. As most studies conducted on this topic, this one is observational, which limits the ability to make inferences about causality. Therefore, we aimed to provide supporting evidence of a causal relationship between OC use and depression through the examination of familial confounding in sister pairs (Li *et al.*, 2020).

## Methods

### Study population

UKB is a population-based cohort that recruited 500,000 participants, aged 37–71 years, from across the United Kingdom (UK) between 2006 and 2010. The study collected extensive data from questionnaires, interviews, physical health measures, biological samples, and imaging. Participants are also linked to health records, including hospital inpatient data, primary care data, cancer, and death registry data. In the present study, we included all female participants of UKB (*N* = 264,557).

### Assessment of exposure

**Figure 1.**
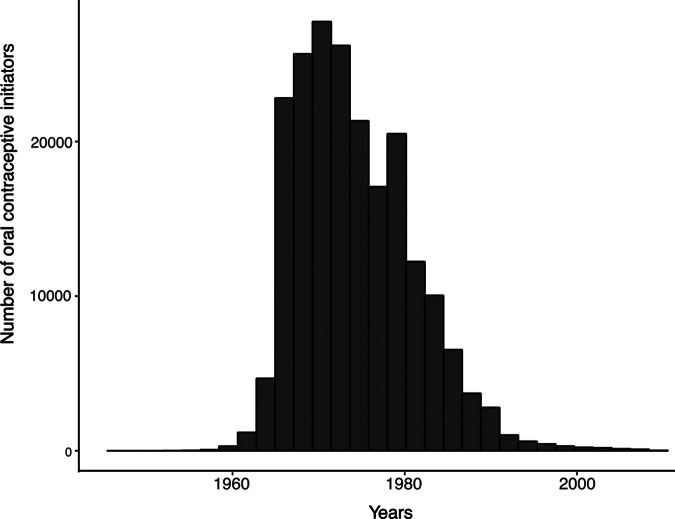
Year of initiating oral contraceptive use. The histogram shows the number of women who first initiated oral contraceptive use each year. The numbers are based on the 205,858 women in the UK Biobank who reported their age at the time of initiation. The year at initiation has been calculated using information on year of birth.

During the initial assessment visit, information on OC use, including the age when first initiated and last discontinued, was obtained through a touch screen questionnaire. The relevant UKB data fields include 2784 (ever taken OCs), 2794 (age started OCs) and 2804 (age when last used OCs). The majority of women in UKB initiated OC during the 1970s/beginning of the 1980s (Fig. 1). During this period, the second-generation pills were predominantly used in the United Kingdom. At the end of the 1960s, OCs that contained levonorgestrel with dosages ranging from 100 to 150 μg, in combination with 20, 30, or 50 μg of ethinyl estradiol, were introduced to the market (Dhont, 2010). For women who were still using OCs (*N* = 4,766), age of last use was set to the age at assessment. Women unsure about OC discontinuation (*N* = 16,223), were excluded in the time-dependent analysis

### Assessment of depression and covariates

Incident depression was addressed as the first diagnosis of depression (Table S1). This information was obtained from either the verbal interview during the assessment at the clinic or the International Classification of Disease code F32 recorded in the inpatient hospital or primary care data, as described in more detail in the Supplementary material and on the UKB website (UK Biobank, 2019). In order to select suitable confounders for our main analysis, we applied the directed acyclic graph approach (VanderWeele *et al.*, 2008) (see Figure S1). Information on potential confounders was assessed from data obtained during the initial assessment center visit and included year of birth, Townsend deprivation index (TDI; used as a proxy for socioeconomic status), number of live births, number of stillbirths, polycystic ovary syndrome (PCOS), age at menarche, age at sexual debut, and family history of severe depression (defined as having one or more first-degree relatives with depression). Adjustment for potential population stratification was achieved by including the first five genetic principal components, as described in the Supplementary methods. For details of each covariate identified in UKB, see Table S2.

### Alternative outcome assessment

To account for women who may have experienced depression but did not seek treatment or receive a diagnosis, we conducted a secondary outcome analysis on the subset of women who completed the UKB mental health questionnaire (MHQ: Table S3) (Davis *et al.*, 2020). The MHQ was designed to assess lifetime depressive disorder using the Composite International Diagnostic Interview Short Form. Relevant data fields from the UKB MHQ include the following: 20446 (ever had prolonged feelings of sadness or depression), 20441 (ever had prolonged loss of interest in normal activities) and 20433 (age at first episode of depressive symptoms). Participants who answered “yes” to either 20446 or 20441 were asked to report their age at the onset of symptoms (20433).

### Statistical analysis

Women were followed from birth until the first occurrence of depression or until the end of follow-up (age at initial UKB assessment visit), whichever came first. OC use was modelled as a time-varying variable using Cox modelling for counting processes (Therneau and Grambsch, 2000). The reference group comprised never users, defined as those who never used OCs, and non-exposed users prior to OC initiation. In the main analyses, we estimated the associated risk of depression within 2 years after OC initiation, in all women, as well as in groups stratified by age at initiation: (1) adolescents (women who initiated OCs before or at their 20th birthday) and (2) adults (women who initiated OCs at age 20 or older). Here the time-varying OC use exposure was coded as “never use” in all women from birth and changed into “initial use” at age of initiation for women who initiated OCs. After 2 years of use, the women were censored (see Supplementary method for more information). In addition, we estimated the lifetime risk of depression, with the time-varying exposure coded as “never use” for all women from birth. This exposure status changed to “ever use” if a woman initiated OC use and continued to be classified as “ever use”, regardless of the age at discontinuation. While age was analyzed as the primary time scale, year of birth was included as a covariate in the models (Cologne *et al.*, 2012) to account for cohort effects. To include only women in their reproductive years, women were censored if they reached menopause, underwent a hysterectomy, or bilateral oophorectomy, whichever came first. We estimated the hazard (rate) ratio (HR) of incident depression among users versus never users and its 95% confidence interval (CI). All analyses were performed using R version 4.1.1.

### Time-dependent analysis

Apart from the main analyses, we conducted additional time-dependent analyses to compare the effect of OC use at initiation, to the effect (i) during remaining years of use, (ii) among recent users, as well as (iii) in previous users. The time-varying exposure to OC use was coded as “never use” for all women from birth and changed to “initial use” when a woman started using OCs. After 2 years of use, the OC exposure variable was reclassified as “remaining years of use”, followed by “recent use” and “previous use” (see Supplementary material).

### Sensitivity analyses

Sensitivity analyses were performed to test whether the OC-associated depression risk remained similar when (1) parous women were censored 1 year before their first live birth to avoid the possible influence of postpartum depression, (2) restricting the sample to women who identified as “white Irish”, “white British” or “other white” (*N* = 257,185) to minimize the risk of confounding due to population stratification, (3) excluding women with other psychiatric disorders or medical indications for OC use to ensure that incident depression was identified and to reduce the risk of confounding by indication and (4) limiting the analysis only to OC users to eliminate the possibility that never users of OC differ from users in ways that may affect disease risk. Specifically, we compared the hazard rates within 2 years after initiation of OC use with those before initiation.

**Table 1.** Distribution of general characteristics in oral contraceptive initiators and never users

	Oral contraceptive initiators (ever users)	Never users^a^
Number (%)	205,860 (80.6)	49,645 (19.4)
Depression, *N* (%)	20,454 (9.94)	4,296 (8.65)
Year of birth, median (full range)	1,952 (1936−1970)	1,946 (1936−1970)
Age, median (1st–3rd quartile)	56 (49−62)	62 (56−66)
TDI, median (1st–3rd quartile)	−2.22 (−3.68 to 0.3)	−1.99 (−3.54 to 0.86)
Number of live births, median (1st–3rd quartile)	2 (1−2)	2 (1−3)
Age at first birth, median (1st–3rd quartile)	26 (22−29)	25 (22−28)
Age of primiparous women at birth of child, median (1st–3rd quartile)	29 (24−34)	28 (24−32)
Number of still births, *N* (%)	4,744 (0.02)	1,693 (0.03)
Polycystic ovary syndrome, *N* (%)	1,603 (0.78)	291 (0.59)
Age at menarche, median (1st–3rd quartile)	13 (12−14)	13 (12−14)
Age at sexual debut, median (1st–3rd quartile)	18 (17−20)	20 (18−23)
Age at menopause, median (1st–3rd quartile)	50 (45−52)	50 (45−53)
Family history of depression, *N* (%)	229 (0.11)	115 (0.23)
Postmenopausal – Yes, *N* (%)	119,231 (57.92)	35,470 (71.45)
Postmenopausal – No, *N* (%)	53,568 (26.02)	6,986 (14.07)
Postmenopausal – Not sure hysterectomy, *N* (%)	23,175 (11.26)	5,889 (11.86)
Postmenopausal – Not sure other, N (%)	9,777 (4.75)	1,168 (2.35)
Age when initiated oral contraceptives, median (1st–3rd quartile)	21 (18−24)	NA
Age when discontinued oral contraceptives, median (1st–3rd quartile)	32 (27−40)	NA
Duration of oral contraceptive use, median (1st–3rd quartile)	10 (5−18)	NA

a Never users are defined as those who never initiated oral contraceptives during the study period (from birth until the recruitment visit).

### Sibling analysis

To assess the possible causal relationship between OC use and depression, we analyzed a subcohort of female siblings in UKB (see Supplementary methods identification details). Inference about Causation from Examination of Familial Confounding is a regression-based approach for determining causality through the use of paired observational data collected from related individuals (Li *et al.*, 2020). The statistical model considers both direct and indirect causes between the exposure and the outcome, as well as the impact of shared familial factors. If there is an association between a person’s outcome and the person’s own exposure that remains unchanged after adjusting for their relative’s exposure, this would indicate a cause-and-effect relationship between the exposure and the outcome. On the other hand, if the association between a person’s outcome and their own exposure, as well as the association between the person’s outcome and the exposure of their relative, both are attenuated towards the null after adjusting for each other, this would not support the existence of a direct causal relationship between the exposure and the outcome. We examined two causal situations: (1) OC use (*X*) and depression (*Y*) are associated due to familial confounding only and (2) *X* and *Y* are associated due to a causal effect of *X* on *Y.* For technical details on the method, see Supplementary methods.

## Results

The study population comprise a total of 264,557 women. Among the women included, 80.6% were ever users. The median time from first initiation to last use of OC use was 10 years, and the median age at initiating and discontinuing use was 21 and 32 years, respectively. At the initial recruitment visit, the ever users were younger, had a lower TDI (higher socioeconomic status), had less often a family history of depression, and had an earlier sexual debut, compared to the never users. During follow-up, a total of 24,750 women received a diagnosis of depression. For participant characteristics, see Table 1.

### OC use and depression

**Figure 2.**
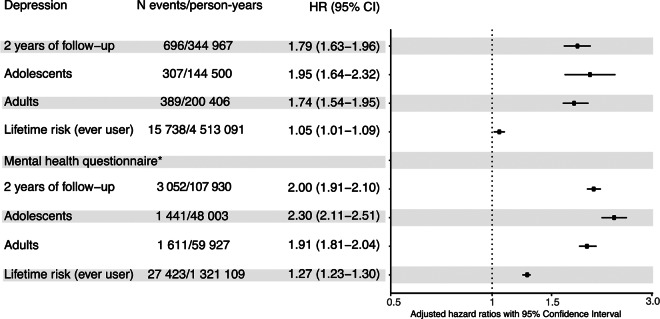
Short-term and lifetime HR of initiation of oral contraceptive use on depression. All estimates are adjusted for year of birth, TDI (used as proxy for socioeconomic status), number of live births, number of still births, PCOS (defined as having ICD10 code E28), age at menarche, age at sexual debut and family history of severe depression (defined as having one or more first-degree relatives with depression). Adolescents are defined as women who initiated oral contraceptives either before or at the age of 20, while adults are defined as women initiating oral contraceptives after turning 20 years old. Lifetime risk = among all women who initiated oral contraceptives at some time point during the follow-up. *Secondary outcome measurement on the subcohort of women (*N* = 82,232) who answered in mental health questionnaire.

During the first 2 years of OC use, there was an increased rate of depression (HR = 1.79, 95% CI: 1.63–1.96), compared with never users (Fig. 2 and Table S4). In the age-stratified analyses, adolescents had an increased rate of depression (HR = 1.95, 95% CI: 1.64–2.32) 2 years following initiation, adults also experienced an increased rate (HR = 1.74, 1.54–1.95: Fig. 2 and Table S5). Although not as pronounced as close to the initiation, also the lifetime risk of depression was higher (HR = 1.05, 95% CI: 1.01–1.09) among ever users compared to never users (Fig. 2 and Table S6).

To capture women with symptoms of depression, which might not have come to clinical attention, we analyzed the subcohort of 82,232 women who completed the online MHQ. Of those, 44,605 reported experiencing at least one of the core depressive symptoms. OC initiation was associated with an increased hazard rate of depressive symptoms (HR = 2.00, 95% CI: 1.91–2.10 during the first 2 years) compared to never users (Fig. 2 and Table S4). OC initiators who completed the online MHQ and began using OC before or at the age of 20 had 130% higher rate of depressive symptoms (HR = 2.30, 95% CI: 2.11–2.51), compared to never users, while the corresponding increase in adult initiators was 92% (HR = 1.92, 1.81–2.04: Fig. 2 and Table S5). Ever use of OC was also associated with an increased rate of depressive symptoms (HR = 1.27, 95% CI: 1.23–1.30) compared with never users (Fig. 2 and Table S6).

### Time-dependent analysis

**Figure 3.**
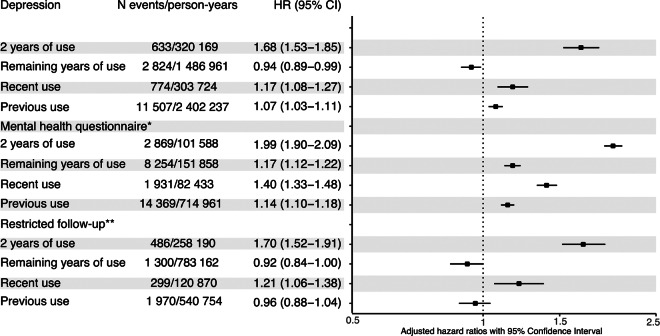
Time-dependent HR of oral contraceptive use and depression. All estimates are adjusted for year of birth, TDI (used as proxy for socioeconomic status), number of live births, number of still births, PCOS (defined as having ICD10 code E28), age at menarche, age at sexual debut and family history of severe depression (defined as having one or more first-degree relatives with depression). *Recent users defined as those who discontinued within 2 years. *Secondary outcome measurement on the subcohort of women (*N* = 82,232) who answered the mental health questionnaire. **Parous women were followed until 1 year before delivery.

In the time-dependent analysis, continued use of OCs was not associated with an increased rate of depression (HR = 0.94, 95% CI: 0.89–0.99: Fig. 3 and Table S7). However, both recent (2 years since cessation) and previous OC users (more than 2 years since cessation) had an increased hazard of depression (HR = 1.17, 95% CI: 1.08–1.27 and 1.07, 1.03–1.11), respectively, compared with never users. The increased hazard of depression in previous users was driven by adolescent OC users (HR = 1.18, 95% CI: 1.12–1.25), whereas no significant association was found for adult OC users 2 years after discontinuing (HR = 1.00, 0.95–1.04: Table S10). Our secondary outcome analysis (MHQ) revealed that even after using OC for more than 2 years, an increase in the hazard rate (HR = 1.13, 95% CI: 1.09–1.17: Fig. 3 and Table S7) was still observed. In line with our primary outcome measure (i.e., a depression diagnosis), an increased association of depressive symptoms was found among recent and previous OC users (HR = 1.40, 95% CI: 1.33–1.48 and 1.13, 1.10–1.17), respectively.

### Sensitivity analyses

Sensitivity analysis on the subcohort of women who self-identified as white did not yield any marked changes to the risk estimates (Tables S4, S6 and S8). Similar estimates were also found when women with a medical indication for OC use and with other psychiatric disorders were excluded (Tables S4 and S9). In the sensitivity analysis where parous women were followed until 1 year before giving birth or depression (whichever came first), the HRs were slightly higher or similar (Fig. 3 and Tables S6–S8). Among those who had ever initiated OCs, the hazard rate of receiving a first depression diagnosis within 2 years after initiation was higher (HR = 1.93, 95% CI: 1.71–2.17: Table S11) than before initiating OCs.

### Sibling analysis

Causal inference analysis was performed on a total of 7,354 first-degree sister pairs. Among these, 81% had initiated OCs. The within-sibling pair correlation for OC use was 0.20 (Pearson’s correlation coefficient, *P* < 0.001), and the corresponding odds ratio (OR) was 3.08 (95% CI: 2.80–3.38). The within-sibling pair correlation for depression was 0.03 (Pearson’s correlation coefficient, *P* = 0.002), and the corresponding OR was 2.16 (1.28–3.40). A sibling’s OC use was positively associated with a depression diagnosis (model 1: *β*_self_ = 0.51, 95% CI: 0.23–0.80: Table S12). The co-sibling’s OC use was also associated with the sibling’s depression diagnosis (model 2: *β*_co-sibling_ = 0.29, 95% CI: 0.001–0.58). Adjusting for the co-sibling’s OC use (model 3), *β′*_self_ remained unchanged (*β′*_self_ = 0.48, 95% CI: 0.19–0.76) compared with *β*_self_ in model 1 (*P* for difference 0.16), while *β*′_co-sibling_ = 0.19 (95% CI: −0.10 to 0.48) was attenuated towards null as compared with *β*_co-sibling_ in model 2. These results support the hypothesis of a causal relationship between OC use and depression, such that OC use increases the risk of depression.

## Discussion

In this study, including 264,557 women, we showed that OC use is associated with an increased risk of depression shortly after initiation. The increased risk declined with continued OC use, but the lifetime risk associated with ever OC use remained significantly increased. Our findings are comparable to what was found in a Danish study (Skovlund *et al.*, 2016), which identified that the risk peaked half a year after initiation and declined with continued use. These results could be explained by hormonal fluctuations induced by OC initiation, which can affect women who are particularly sensitive to changes in the levels of hormones and their metabolites, such as allopregnanolone (Hantsoo and Epperson, 2015). These fluctuations could alter GABAergic regulation of the hypothalamic–pituitary–adrenocortical in this group of women (Gordon *et al.*, 2015). Our results are also comparable to what was seen in a study estimating the risk of suicidal behaviour, which was found to be higher during the initial use of OC (Edwards *et al.*, 2022).

Unlike most previous studies, we estimated the time-varying effects of OC use using a “new user” design approach (Yoshida *et al.*, 2015). This allowed us to capture events occurring in different time windows during follow-up. Using a prevalent user design, which assumes the effect is similar in current and new users, would miss the increased risk seen early in the treatment course. This can explain why some previous studies, where the rate of depression among current OC users is compared to the rate among never or previous users, have not identified a significant effect (Cheslack-Postava *et al.*, 2015; Lundin *et al.*, 2022; McKetta and Keyes, 2019). Our study found higher depression rates in the first years after discontinuing OCs. This may reflect that women who get mood-related problems discontinue OC use, but are not diagnosed with depression until after cessation.

Our results, consistent with the Danish study (Skovlund *et al.*, 2016), suggest that the risk of depression is increased not only among adolescents initiating OCs but also among women older than 20 years. However, our findings showed that women who used OCs during adolescence remained at a heightened risk even after they discontinued, whereas such a risk was not apparent among adult OC users. It has been hypothesized that the increased risk later in life among those who used OCs during adolescence may be attributed to a greater susceptibility to gonadal hormones, including hormonal contraception, during crucial developmental periods that affect the organization of brain structures and may lead to long-lasting changes (Anderl *et al.*, 2022, 2020; Cahill, 2018; de Wit *et al.*, 2020).

Residual confounding, due to familial disposition, early menarche (Karina and Sivakumaran, 2017) and sexual debut (McKetta and Keyes, 2019), has been suggested to explain the increased risk of depression associated with OC use. These factors were all adjusted for in the current study. However, other potential confounders include medical indications for hormonal contraception use (Duke *et al.*, 2007). The present study accounted for medical indication by excluding women with dysmenorrhoea, endometriosis, and PCOS, but as the premenstrual dysphoric disorder diagnosis did not exist in the ICD10, it was not possible to directly adjust for this. However, the heritability of premenstrual dysphoric disorder is high (Condon, 1993), and therefore the sibling analysis should partly account for any such confounding.

Our findings must be interpreted in the light of several limitations. First, the main limitation of this study is the potential recall bias in the self-reported data, particularly regarding the age of OC use initiation and discontinuation. Second, the study is subject to a certain sample selection bias as the UKB consists of a healthier population compared to the general population of the UK (Fry *et al.*, 2017), which hampers the generalizability of our findings. In addition, white Europeans are overrepresented in the UKB. The small number of observations in non-white participants precluded us from performing analyses stratified by ethnic background. Third, we were not able to evaluate different formulations or routes of administration as we did not have detailed information on the OC type used. Hence, our results might not be generalizable to all types of OCs used today. Nonetheless, given the birth year of the women included in the study, our results are mainly based on the second-generation OCs containing a combination of both estrogen and progesterone, which are still used by many women today. Fourth, as we only had information about age at first and last use, we were not able to capture if they stopped and restarted in between, which makes the time-dependent association less precise. Fifth, there is a potential for recall bias also for the self-reported family history data used as covariate information. However, missing family history data is minimal (88% report parental history and 93% report sibling history) (Hujoel *et al.*, 2022), and it has been shown that self-reported family history is accurate (∼80% correlation between true and self-reported family history, based on sibling concordance) (Hujoel *et al.*, 2020). Last, some confounders were only measured once, which could impact our estimates. However, we incorporated time-varying covariates when possible to account for changes during follow-up.

## Conclusion

Our findings support that OC use is causally associated with an increased risk of depression in adolescents as well as in adults, especially shortly after the initiation. It is important to emphasize that most women tolerate OCs well without experiencing adverse mood effects, making them a great option for many. However, educating OC users, screening for depression, informing primary healthcare practitioners regarding the OC–depression relationship and conducting further research to determine the cause of hormone contraceptive-precipitated depression are warranted.

## Data Availability

The data used for this study is available for bona fide researchers from the UK Biobank Resource (http://www.ukbiobank.ac.uk/about-biobank-uk/) and can be accessed by an application to the UK Biobank.

## Supplementary material

For supplementary material accompanying this paper visit http://doi.org/10.1017/S2045796023000525. click here to view supplementary material
