# Supplementary material for: Population-based cohort study of oral contraceptive use and risk of depression
Source: Epidemiol Psychiatr Sci. 2023 Jun 12;32:e39. doi: 10.1017/S2045796023000525 (PMC10294242; doi:10.1017/S2045796023000525)
Supplement: Supplementary file 1 [file epssup.zip › S2045796023000525sup002.docx]

SUPPLEMENTARY MATERIAL

**Population-based cohort study of oral contraceptive use and risk of depression**

Therese Johansson, MSc^1,2^, Søren Vinther Larsen, MD^3,4^, Minh Bui, PhD^5^, Weronica E. Ek, PhD^1^, Torgny Karlsson, PhD^1^, Åsa Johansson, PhD^1^

^1^Department of Immunology, Genetics and Pathology, Science for Life Laboratory, Uppsala University, Sweden

^2^Centre for Women’s Mental Health during the Reproductive Lifespan – Womher, Uppsala University, Sweden

^3^Neurobiology Research Unit, Copenhagen University Hospital, Rigshospitalet, Denmark

^4^Department of Clinical Medicine, University of Copenhagen, Denmark

^5^Centre for Epidemiology and Biostatistics, Melbourne School of Population and Global Health, University of Melbourne, Melbourne, VIC, Australia

**Study population**

**UK Biobank**

The UK Biobank (UKB) is the world’s largest prospective population-based cohort with information on clinical and demographic variables in combination with genetics, metabolites, and biomarkers. Before recruitment, invitations were sent out to approximately 10 million UK residents, aged 40-69 years, living within 10 miles from any of the 27 assessment clinics across the UK. A total of 502,617 people volunteered to participate and the first assessments took place between 2006-2010 (Allen *et al.*, 2012). Two of the main sources of bias due to the process of selection within the UKB are 1) participation bias, which was reduced by offering incentives and by tracking non-responders to encourage participation, and 2) healthy-volunteer selection bias, which was also reduced by offering incentives and reaching out to participants with different health status, including those with chronic conditions (Bycroft *et al.*, 2018).

**Depression outcomes**

Identification of first incident depression was obtained from the “first occurrence” UKB data field: 130894, which includes diagnoses recorded before and after the initial assessment visit. These data were generated by mapping the clinical codes from primary care, hospital inpatient admissions, death record, and self-reported medical conditions to the 3-character International Classification of Disease (ICD-10) code ‘F32’. For each participant, the data field provides the earliest date at which a depression diagnosis was identified in any of the sources. The self-reported medical conditions were derived from data field 20008 which includes the interpolated year when a non-cancer illness was first diagnosed. The self-reported medical conditions were obtained during the initial assessment visit where participants were asked by a trained nurse whether they had ever been told by a doctor that they had an illness or disability. To record an illness the interviewer showed a tree structure of diagnoses (organized by system and based loosely on the ICD-10 codes). For each illness the participant recorded, the interviewer recorded the date of the first diagnosis. If the participant couldn’t recall the date but could give an approximation, it was preferable to provide an estimation rather than enter unknown. They could provide either their age at diagnosis or the year diagnosis occurred. If a participant reported an age, the value presented is the fractional year corresponding to the mid-point of that age. For example, if a participant reported the first depressive episode at age 30, then the value is the date at which she/he was 30 years + 6 months. If the participant gave a calendar year, e.g., 1970, the value presented is half-way through that year 1970.5. If a participant had both an ICD10 code identified in the registers (i.e., data from either the death register, hospital inpatient admissions, or primary care) and a self-reported medical condition, the date corresponding to the earliest date was used. Further information on how the “first occurrence” data were derived is detailed in the document “First Occurrence of Health Outcomes Defined by 3-character ICD10 Code”(UK Biobank, 2019). For information on the number of incident depression identified in the different sources, see **Supplementary Table S1**.

**Mental health questionnaire**. Self-report of medical conditions and data from record linkages might capture more severe cases with less variance. Therefore, we conducted sensitivity analyses on the subcohort of women answering the online mental health questionnaire (MHQ) in (UKB) (Davis *et al.*, 2020), to include those women who never sought or received treatment and therefore were not captured in the “First occurrence” data fields. An expert group designed the MHQ which was sent out to all UKB participants. By August 2017 a total of 157,366 participants had completed the questionnaire, and the data became available. The core of the MHQ was the measurement of lifetime depressive disorder using the Composite International Diagnostic Interview Short Form (CIDI-SF). The relevant UKB data fields include: 20446 (ever had prolonged feelings of sadness or depression), 20441 (ever had prolonged loss of interest in normal activities), and 20433 (age at first episode of depression). If a participant had answered yes on either 20446 or 20441, they were further asked how old they were when this episode first occurred (20433). Controls were those who answered “No” on both 20441 and 20446. For information on the number of comorbid first incident depression diagnoses identified in the main outcome (“first occurrence” data field) and in the MHQ see **Supplementary Table S3**.

**Covariates**

Information on the year of birth and Townsend Deprivation Index (TDI - used as a proxy for socioeconomic status), were obtained from the local National Health Service Primary Care Trust register prior to participation in the UKB. Information on menopausal status was obtained during the initial visit. Women were asked via a touchscreen questionnaire: “Have you had your menopause (periods stopped)?”. The answer options were “yes”, “no”, “not sure – had a hysterectomy”, “not sure – other reason” and “prefer not to answer”. Participants who had answered “prefer not to answer” were excluded from all analyses that included menopausal status. Family history of medical conditions was assessed from the touchscreen questionnaire and participants with a first-degree relative suffering from severe depression was classified as having a family history of depression. Age at menarche (period started), age at sexual debut, age at menopause (last menstrual cycle), number of live births, number of stillbirths, age at first live birth among multiparous women, and the age at which the primiparous women gave birth were assessed from the touchscreen questionnaire completed at the initial visit to the assessment center. Participants answering “do not know” or “prefer not to answer” were excluded from all analyses.

In the analyses including all women independent of ethnic background, the five first genetic principal components (PCs) were included as covariates in the model, to adjust for confounding due to population stratification. This is a state-of-the-art method, widely used in genetic epidemiology, to reduce the effect of confounding due to co-incidental geographic/cultural variation in the exposure and the outcome, and it has been shown that also within the UK Biobank there are structures that can have implications for epidemiological studies (Haworth *et al.*, 2019). The genetic PCs in the UK Biobank are computed based on the genetic kinship (pairwise genetic distance) between the individuals of the cohort as has been described previously (Bycroft *et al.*, 2018).

**Time-varying covariates.** Confounders with information on changes during the follow-up time were incorporated in the model as time-varying covariates. The inclusion of time-varying covariates was achieved by applying the counting process approach (Therneau and Grambsch, 2000). In this approach, the data for each individual are divided into multiple episodes, such that no covariate is allowed to change in value within an episode. Hence, all covariates can be treated as time-fixed within each episode. For example, a woman who reached menarche at age 13 and received a depression diagnosis at age 25 will be assigned a record of two lines in the data frame: the first line represents the “state” of the individual before menarche (0, 13], while the second line represents the “state” after menarche (13, 25], assuming all other variables remain constant during this time. To arrange and structure data with multiple records for each individual, the tmerge function in the R package survival (v 2.44.1.1) was used.

**Time-varying exposure.** Time-varying exposure effects were also modeled by the counting process (Therneau and Grambsch, 2000), in the same way as described above for time-varying covariates. Age was used as the primary time scale to allow for a natural nonparametric adjustment of age in the Cox model and follow-up started at age 0. To investigate whether there is a difference in risk associated with length of use, we allowed the exposure variable to change value at initiation, after two years of use, at discontinuation, and one year after the discontinuation. This was accomplished using the same principle as for the time-varying covariates. We defined a variable, tgroup, with the following states: tgroup = 1 represents the first time interval, in which an individual is classified as a non-user, tgroup = 2 represents the second time interval, in which the individual has been a user for <2 years, tgroup = 3 corresponds to a time interval of >2 years of use until age at discontinuation (remaining years of use), tgroup = 4 corresponds to recent use, defined as <2 years since discontinuation of use and tgroup = 5 corresponds to previous users (>2 years since cessation). This approach correctly characterizes the exposure status and classifies the person-time of the user before oral contraceptive initiation as unexposed follow-up time, aiming to avoid immortal time bias, an otherwise common bias in survival analysis.

In all analyses, “never users” were used as the reference group in an attempt to reduce healthy-user bias. Two years after cessation, a woman was therefore classified as a previous user (tgroup = 5) instead of being classified a “non-user”. If women who discontinue oral contraceptives are depressive sensitive, results might be underestimated when “non-users” are used as reference. Note that the “never user” group will include a larger proportion of women of younger age (never users + users from birth until the age at first use). Since few women develop depression during childhood, a low event rate will be observed in the “never users” group. However, since age is used as the primary time-scale in our analysis, the Hazard Rate (HR) compares the rate among women of the same age. Therefore, the variation in event rate with age does not bias the HR.

Age at first depression diagnosis was calculated based on the difference in years between the date of depression diagnosis and the date of birth and rounded downwards to enable comparison with e.g., age at initiation of oral contraceptive use. Depression diagnoses that occurred between the age at initiation and the age at discontinuation were considered as events during use. All Cox regression models were fitted using the coxph function in the survival package (v 2.44.1.1)

**Inference about causation from examination of familial confounding (ICE FALCON)**

To explain the method in our study (Li, Bui and Hopper, 2020), oral contraceptive use is the exposure (X) and depression the outcome (Y), ‘self’ refers to an individual, and ‘co-sibling’ refer to the individual’s sibling. Given that, oral contraceptive use and depression are positively associated within an individual (X_self_ and Y_self_), there must be familial (genetic or shared environment) factors that determine oral contraceptive use, and familial factors that determine depression status. If there is also a ‘cross-trait cross-pair’ association (between Y_self_ and X_co-sibling_ or between X_self_ and Y_co-sibling_), such that oral contraceptive use of a sibling is associated with depression of her co-sibling, this could be due to: (i) the effects of familial confounders, (ii) causation between X and Y, provided that X_self_ and X_co-sibling_ are correlated due to S_X_ (the combination of unmeasured causes, genetic and/or non-genetic, that influence X only in both siblings, and/or that Y_self_ and Y_co-sibling_ are correlated due to S_Y_ (the combination of unmeasured causes genetic and/or non-genetic that influence Y only in both siblings_,_ or (iii) a combination of both (i) and (ii). Three Cox regression models for the hazard rate h(t|**X**)=h_0_(t)*exp**(beta**^T^***X)** were fitted, such that:

$$Model 1: \boldsymbol{\beta}^{T}\boldsymbol{X}= \beta selfXself + \boldsymbol{\beta}_{Cov}^{T}\boldsymbol{X}_{Cov}$$

$$Model 2: \boldsymbol{\beta}^{T}\boldsymbol{X}= \beta cosiblingXcosibling+ \boldsymbol{\beta}_{Cov}^{T}\boldsymbol{X}_{Cov}$$

$$Model 3: \boldsymbol{\beta}^{T}\boldsymbol{X}= \beta^{'}selfXself+ \beta'cosiblingXcosibling+ \boldsymbol{\beta}_{Cov}^{T}\boldsymbol{X}_{Cov}$$

If the association is due to familial confounding only, there will be an association between Y and X within an individual $(\beta$_self_, Model 1), and a ‘cross-trait cross-pair association’ $(\beta$_co-sibling_, Model 2).When both within-individual (X_self_) and cross-trait (X_co-sibling_) terms are included (Model 3), $\beta'$_self_ and $\beta'$_co-sibling_ will both be attenuated with similar magnitude toward the null compared to $\beta$_self_ and $\beta$_co-sibling_.

If there is a causal effect from oral contraceptive use to depression only, there will be a within-individual and cross-trait cross-pair association ($\beta$_self_, Model 1 and $\beta$_co-sibling_, Model 2). Adjusting for X_self_ (Model 3), the association between Y_self_ and X_co-sibling_ ($\beta'$_co-sibling_) will be attenuated to the null. Conversely, adjusting for X_co-sibling_ (Model 3), the conditional association between Y_self_ and X_self_ will be similar to the within-association ($\beta$_self_, Model 1).

We assessed the statistical significance of the change in regression coefficients using a nonparametric bootstrap method. 500 bootstrap datasets were generated. For each bootstrap dataset, ICE FALCON was applied to calculate the difference in regression coefficients, e.g., between $\beta$_self_ and $\beta'$_self_. The standard error of the null was taken as the standard deviation of the difference across the 500 generated datasets. We estimated the HR during oral contraceptive use with follow-up time as the primary time scale, defined as the time between age at initiation and discontinuation. Women who had a first depression diagnosis prior to the oral contraceptive initiation were excluded from the analyses, while diagnoses between initiation and discontinuation were considered as events. For each never user, the age at start and end of follow-up were selected at random from an ever user with matched age at the initial assessment to UKB. Cox proportional hazard modelling with robust standard-error estimates was applied by adding a cluster(id) term in the model to account for related individuals in our dataset. We also adjusted for the five first PCs, TDI (used as a proxy for socioeconomic status), and age at entry.

**Identification of sibling pairs**

Siblings or other close relationships among UKB participants were not recorded during the assessment of phenotypic information. However, UKB conducted a relatedness analysis (Bycroft *et al.*, 2018) on all participants using the KING software to calculate the kinship coefficient and the estimate of identity-by-state (IBS0). A total of 107,162 related pairs were identified with their corresponding kinship coefficient and IBS0.

In order to identify sibling pairs in our study population, we applied the same filters used by (Bycroft *et al.*, 2018). Sibling and parent-child relationships have the same expected relatedness coefficient (genome sharing) of 0.25 but can be distinguished by their fraction of markers for which they share no alleles (IBS0). Parent-child relationships are distinguished by those with IBS0 < 0.0012. Thus, sister pairs were identified by selecting those with IBS0 > 0.0012 and a kinship coefficient > 0.176, yielding a set of 7,465 pairs of participants to which ICE FALCON was applied.

Figure S1. Directed Acyclic Graph (DAG) on the association between oral contraceptive use and depression among female participants in UK Biobank.

References

Allen, N. *et al.* (2012) ‘UK Biobank: Current status and what it means for epidemiology’, *Health Policy and Technology*. Elsevier, 1(3), pp. 123–126. doi: 10.1016/J.HLPT.2012.07.003.

Bycroft, C. *et al.* (2018) ‘The UK Biobank resource with deep phenotyping and genomic data’, *Nature*, 562(7726), pp. 203–209. doi: 10.1038/s41586-018-0579-z.

Davis, K. A. S. *et al.* (2020) ‘Mental health in UK Biobank – development, implementation and results from an online questionnaire completed by 157 366 participants: a reanalysis’, *BJPsych Open*. 2020/02/06. Cambridge University Press, 6(2), p. e18. doi: DOI: 10.1192/bjo.2019.100.

Haworth, S. *et al.* (2019) ‘Apparent latent structure within the UK Biobank sample has implications for epidemiological analysis’, *Nature Communications*, 10(1), p. 333. doi: 10.1038/s41467-018-08219-1.

Li, S., Bui, M. and Hopper, J. L. (2020) ‘Inference about causation from examination of familial confounding (ICE FALCON): a model for assessing causation analogous to Mendelian randomization’, *International Journal of Epidemiology*, 49(4), pp. 1259–1269. doi: 10.1093/ije/dyaa065.

Therneau, T. M. and Grambsch, P. M. (2000) *Modeling Survival Data: Extending the Cox Model*.

UK Biobank (2019) *First Occurrence of Health Outcomes Defined by 3-character ICD10 codeNo Title*. Available at: https://biobank.ndph.ox.ac.uk/showcase/ukb/docs/first_occurrences_outcomes.pdf.
