# Supplementary material for: Population-based cohort study of oral contraceptive use and risk of depression
Source: Epidemiol Psychiatr Sci. 2023 Jun 12;32:e39. doi: 10.1017/S2045796023000525 (PMC10294242; doi:10.1017/S2045796023000525)
Supplement: Supplementary file 1 [file epssup.zip › S2045796023000525sup003.docx]

SUPPLEMENTARY TABLES

**Population-based cohort study of oral contraceptive use and risk of depression**

Therese Johansson, MSc^1,2^, Søren Vinther Larsen, MD^3.4^, Minh Bui, PhD^5^, Weronica E. Ek, PhD^1^, Torgny Karlsson, PhD^1^, Åsa Johansson, PhD^1^

^1^Department of Immunology, Genetics and Pathology, Science for Life Laboratory, Uppsala University, Sweden

^2^Centre for Women’s Mental Health during the Reproductive Lifespan – Womher, Uppsala University, Sweden

^3^Neurobiology Research Unit, Copenhagen University Hospital, Rigshospitalet, Denmark

^4^Department of Clinical Medicine, University of Copenhagen, Denmark

^5^Centre for Epidemiology and Biostatistics, Melbourne School of Population and Global Health, University of Melbourne, Melbourne, VIC, Australia

Supplementary tables

Table S1. Identification of first incident depression in the study population.

Table S2. Identification of exposure, outcome, covariates, and exclusion variables in the UK Biobank database

Table S3. Overlapping incident depression diagnoses identified via “First occurrence” data field and the mental health questionnaire

Table S4. Risk of depression close to initiation of oral contraceptive use (2 years of follow-up)

Table S5. Risk of depression close to initiation of oral contraceptive use (2 years of follow-up) among adolescents and adults

Table S6. Life-time risk of oral contraceptive use

Table S7. Time-dependent effects of oral contraceptive use

Table S8. Time-dependent effects of oral contraceptive use among white participants

Table S9. Time-dependent effects of oral contraceptive use excluding women with a medical indication for oral contraceptive use and other psychiatric disorders

Table S10. Time-dependent effects of oral contraceptive use in adolescence and adults separately

Table S11. Ever users only analysis

Table S12. ICE FALCON sensitivity analysis

Table S1. Identification of first incident depression in the study population

|  | Data-fields | N total (N without a date) | N Primary  care only | N primary care and other source(s) | N Hospital  admissions  data only | N Hospital admissions data and other source(s) | N Self-report only | N Self-report and other source(s) |
| --- | --- | --- | --- | --- | --- | --- | --- | --- |
| First incidence of  depression identified in  “First occurrence” data fields* | 130894/  130895 | 25 800 (7) | 4 893 | 1 708 | 1 067 | 170 | 10 884 | 7 058 |

*The outcome used in the main analysis

Table S2. Identification of exposure, outcome, covariates, and exclusion variables in the UK Biobank database

| Covariates | Data-fields | Origin | N* |
| --- | --- | --- | --- |
| Depression-Yes | 130894 | Registers and Self-reported | 25773 |
| Depression-No | 130894 | Registers and Self-reported | 238784 |
| Year of birth | 34 | Registry | 264557 |
| Age | 21003 | Registry | 264557 |
| TDI (Townsend deprivation index) | 189 | Derived variable | 264243 |
| Number of live births | 2734 | Self-reported | 263957 |
| Age at first birth | 2754 | Self-reported | 214697 |
| Age of primiparous women at birth of child | 3872 | Self-reported | 35231 |
| Number of still births | 3829 | Self-reported | 264304 |
| Polycystic ovary syndrome-Yes | 130736 | Registers and Self-reported | 1957 |
| Polycystic ovary syndrome-No | 130736 | Registers and Self-reported | 254557 |
| Age at menarche (when period started) | 2714 | Self-reported | 256307 |
| Age first had sexual intercourse | 2139 | Self-reported | 230603 |
| Age at menopause (last menstrual period) | 3581 | Self-reported | 192830 |
| Family history of severe depression-Yes | 20107/10/11 | Self-reported | 38731 |
| Family history of severe depression-No | 20107/10/11 | Self-reported | 225417 |
| Post-menopausal-No | 2724 | Self-reported | 62049 |
| Post-menopausal-Yes | 2724 | Self-reported | 160314 |
| Post-menopausal-Not sure hysterectomy** | 2724 | Self-reported | 30165 |
| Post-menopausal-Not sure, other** | 2724 | Self-reported | 11302 |
| Age when initiated OC | 2794 | Self-reported | 205858 |
| Age when discontinued OC | 2804 | Self-reported | 189635 |
| Age at first depressive symptoms episode*** | 20433 | Self-reported | 44605 |
| Dysmenorrhea-Yes | 132151 | Registers and Self-reported | 11897 |
| Dysmenorrhea-No | 132151 | Registers and Self-reported | 252660 |
| Endometriosis-Yes | 132123 | Registers and Self-reported | 9657 |
| Endometriosis-No | 132123 | Registers and Self-reported | 254900 |
| Bipolar affective disorder-Yes | 130893 | Registers and Self-reported | 1131 |
| Bipolar affective disorder-No | 130893 | Registers and Self-reported | 263429 |
| Schizophrenia-Yes | 130875 | Registers and Self-reported | 481 |
| Schizophrenia-No | 130875 | Registers and Self-reported | 264076 |
| Obsessive-compulsive disorder-Yes | 130909 | Registers and Self-reported | 490 |
| Obsessive-compulsive disorder-No | 130909 | Registers and Self-reported | 264067 |
| Posttraumatic stress disorder-Yes | 130911 | Registers and Self-reported | 9316 |
| Posttraumatic stress disorder-No | 130911 | Registers and Self-reported | 255241 |

*N – number of individuals with covariate data available for quantitative variables, and number of individuals within each level for factors. **Participants that were not sure about menopausal status were divided into those that were unsure because of hysterectomy and those unsure for other, unspecified, reasons. ***Women who answered yes on the mental health questionnaire and their age at their first depressive symptom episode.

Table S3. Overlapping incident depression diagnoses identified via “First occurrence” data field and the mental health questionnaire

|  |  | Incident depression identified in the “First occurrence”.  Data fields: 130894/130895* | |
| --- | --- | --- | --- |
|  |  | Yes | No |
| Depressive episode identified via the mental health questionnaire. Data-field: 20433** | Yes | 6 654 | 37 951 |
|  | No | 576 | 37 051 |

*The outcome that was analysed in the main analysis. **The outcome analysed in the secondary anslyes based on the answers from the mental health questionnaire (N = 82 232) included to capture less severe cases with more variance, based on the follow-up questionnaire sent out to all UK Biobank participants. A total of 6 654 out of 44 605 answering age at first depressive episode were also identified in the “First occurrence” data fields.

Table S4. Risk of depression close to initiation of oral contraceptive use (2 years of follow-up)

| Depression (Main outcome) | | | | | | | | |
| --- | --- | --- | --- | --- | --- | --- | --- | --- |
|  | No. of events | Person-Years | Unadjusted HR (95% CI) | P value | No. of events | Person-Years | Adjusted HR (95% CI)* | P value |
| Never user | 6684 | 7264140 | 1 (Reference) |  | 5156 | 5937995 | 1 (Reference) |  |
| 2 years of follow-up | 771 | 387565 | 1.90 (1.76-2.06) | <0.001 | 696 | 344967 | 1.79 (1.63-1.96) | <0.001 |
| Depression risk two years after oral contraceptive initiation among the women answering the mental health questionnaire*** | | | | | | | | |
|  | No. of events | Person-Years | Unadjusted HR (95% CI) | P value | No. of events | Person-Years | Adjusted HR (95% CI)* | P value |
| Never user | 13742 | 2046575 | 1 (Reference) |  | 12176 | 1827443 | 1 (Reference) |  |
| 2 years of follow-up | 3226 | 115911 | 2.21 (2.12-2.31) | <0.001 | 3052 | 107930 | 2.00 (1.91-2.10) | <0.001 |
| Depression risk two years after oral contraceptive initiation among white participants | | | | | | | | |
|  | No. of events | Person-Years | Unadjusted HR (95% CI) | P value | No. of events | Person-Years | Adjusted HR (95% CI)* | P value |
| Never user | 6272 | 6794439 | 1 (Reference) |  | 4925 | 5640523 | 1 (Reference) |  |
| 2 years of follow-up | 747 | 371279 | 1.88 (1.74-2.04) | <0.001 | 678 | 332362 | 1.78 (1.62-1.96) | <0.001 |
| Depression risk two years after oral contraceptive initiation after excluding medical indication for oral contraceptive use**** | | | | | | | | |
|  | No. of events | Person-Years | Unadjusted HR (95% CI) | P value | No. of events | Person-Years | Adjusted HR (95% CI)* | P value |
| Never user | 4545 | 6135308 | 1 (Reference) |  | 3604 | 5076498 | 1 (Reference) |  |
| 2 years of follow-up | 577 | 340333 | 1.90 (1.73-2.09) | <0.001 | 512 | 302630 | 1.82 (1.64-2.03) | <0.001 |

*Adjusted for principal components 1-5, year of birth, Townsend deprivation index, polycystic ovary syndrome, age at menarche, age at sexual debut, and family history of depression. **Women who reported the age at which they initiated oral contraceptives but were uncertain about the age at which they discontinued. ***Assessment of depression from mental health questionnaire. **** Excluding women with a medical indication for oral contraceptive use and other psychiatric disorders (the relevant ICD10 codes include: E28 (ovarian dysfunction), N94 (dysmenorrhea), N80 (endometriosis), F31 (bipolar affective disorder), F20 (schizophrenia), F42 (obsessive-compulsive disorder), F43 (posttraumatic stress disorder).

Table S5. Risk of depression close to initiation of oral contraceptive use (2 years of follow-up) among adolescents and adults

| Depression risk two years after oral contraceptive initiation among adolescents | | | | | | | | |
| --- | --- | --- | --- | --- | --- | --- | --- | --- |
| Depression | No. of events | Person-Years | Unadjusted HR (95% CI) | P value | No. of events | Person-Years | Adjusted HR (95% CI)* | P value |
| Never user | 4618 | 3847613 | 1 (Reference) |  | 3542 | 3097152 | 1 (Reference) |  |
| 2 years of follow-up | 324 | 156250 | 2.49 (2.14-2.89) | <0.001 | 307 | 144500 | 1.95 (1.64-2.32) | <0.001 |
| Depression risk two years after oral contraceptive initiation among adults | | | | | | | | |
| Depression | No. of events | Person-Years | Unadjusted HR (95% CI) | P value | No. of events | Person-Years | Adjusted HR (95% CI)* | P value |
| Never user | 5441 | 5468610 | 1 (Reference) |  | 4266 | 4400667 | 1 (Reference) |  |
| 2 years of follow-up | 447 | 231315 | 1.81 (1.63-2.01) | <0.001 | 389 | 200406 | 1.74 (1.54-1.95) | <0.001 |
| Depression risk two years after oral contraceptive initiation in adolescents among the women answering the mental health questionnaire** | | | | | | | | |
| Depression | No. of events | Person-Years | Unadjusted HR (95% CI) | P value | No. of events | Person-Years | Adjusted HR (95% CI)* | P value |
| Never user | 7905 | 1049533 | 1 (Reference) |  | 6917 | 931523 | 1 (Reference) |  |
| 2 years of follow-up | 1510 | 50644 | 2.74 (2.54-2.96) | <0.001 | 1441 | 48003 | 2.30 (2.11-2.51) | <0.001 |
| Depression risk two years after oral contraceptive initiation in adults among the women answering the mental health questionnaire*** | | | | | | | | |
| Depression | No. of events | Person-Years | Unadjusted HR (95% CI) | P value | No. of events | Person-Years | Adjusted HR (95% CI)* | P value |
| Never user | 10918 | 1483853 | 1 (Reference) |  | 9669 | 1314353 | 1 (Reference) |  |
| 2 years of follow-up | 1716 | 65267 | 2.00 (1.89-2.12) | <0.001 | 1611 | 59927 | 1.92 (1.81-2.04) | <0.001 |

*Adjusted for principal components 1-5 (in the analysis including all women with different ethnic backgrounds), year of birth, Townsend deprivation index, polycystic ovary syndrome, age at menarche, age at sexual debut, and family history of depression. **Women who reported the age at which they initiated oral contraceptives but were uncertain about the age at which they discontinued. ***Assessment of depression from mental health questionnaire. Adolescents are defined as women who initiated oral contraceptives either before or at the age of 20, while adults are defined as women initiating oral contraceptives after turning 20 years old.

Table S6. Life-time risk of oral contraceptive use

| Depression | | | | | | | | |
| --- | --- | --- | --- | --- | --- | --- | --- | --- |
|  | No. of events | Person-Years | Unadjusted HR (95% CI) | P value | No. of events | Person-Years | Adjusted HR (95% CI)* | P value |
| Never user | 6684 | 7264140 | 1 (Reference) |  | 5156 | 5937995 | 1 (Reference) |  |
| Ever user | 17286 | 5006973 | 1.49 (1.44-1.53) | <0.001 | 15738 | 4513091 | 1.05 (1.01-1.09) | <0.001 |
| Mental health questionnaire** | | | | | | | | |
|  | No. of events | Person-Years | Unadjusted HR (95% CI) | P value | No. of events | Person-Years | Adjusted HR (95% CI)* | P value |
| Never user | 13742 | 2046575 | 1 (Reference) |  | 12176 | 1827443 | 1 (Reference) |  |
| Ever user | 28944 | 1416493 | 1.50 (1.46-1.54) | <0.001 | 27423 | 1321109 | 1.27 (1.23-1.30) | <0.001 |
| Restricted follow-up*** | | | | | | | | |
|  | No. of events | Person-Years | Unadjusted HR (95% CI) | P value | No. of events | Person-Years | Adjusted HR (95% CI)* | P value |
| Never user | 3109 | 5796889 | 1 (Reference) |  | 2398 | 4799201 | 1 (Reference) |  |
| Ever user | 4512 | 1983446 | 1.41 (1.34-1.49) | <0.001 | 4090 | 1793550 | 1.05 (0.99-1.11) | 0.11 |
| Lifetime risk of oral contraceptive use among white participants | | | | | | | | |
|  | No. of events | Person-Years | Unadjusted HR (95% CI) | P value | No. of events | Person-Years | Adjusted HR (95% CI)* | P value |
| Never user | 6272 | 6794439 | 1 (Reference) |  | 4925 | 5640523 | 1 (Reference) |  |
| Ever user | 16765 | 4811788 | 1.49 (1.44-1.54) | <0.001 | 15317 | 4357350 | 1.06 (1.01-1.11) | 0.02 |

*Adjusted for principal components 1-5, year of birth, Townsend deprivation index, number of live births, number of stillbirths, polycystic ovary syndrome, age at menarche, age at sexual debut, and family history of depression (excluding the number of live births and stillbirths when follow-up was restricted). **Assessment of depression from mental health questionnaire. ***Parous women were followed up until one year before delivery.

Table S7. Time-dependent effects of oral contraceptive use

| Depression | No. of events | Person-Years | Unadjusted HR (95% CI) | P value | No. of events | Person-Years | Adjusted HR (95% CI)* | P value |
| --- | --- | --- | --- | --- | --- | --- | --- | --- |
| Never user | 6564 | 6905974 | 1 (Reference) |  | 5051 | 5657296 | 1 (Reference) |  |
| 2 years of use | 690 | 355450 | 1.89 (1.74-2.06) | <0.001 | 633 | 320169 | 1.68 (1.53-1.85) | <0.001 |
| Remaining years of use | 3045 | 1630482 | 1.32 (1.56-1.38) | <0.001 | 2824 | 1486961 | 0.94 (0.89-0.99) | 0.02 |
| Recent user** | 833 | 338227 | 1.56 (1.45-1.68) | <0.001 | 774 | 303724 | 1.17 (1.08-1.27) | <0.001 |
| Previous user | 12716 | 2672513 | 1.49 (1.45-1.55) | <0.001 | 11507 | 2402237 | 1.07 (1.03-1.11) | <0.001 |
| Mental health questionnaire*** | No. of events | Person-Years | Unadjusted HR (95% CI) | P value | No. of events | Person-Years | Adjusted HR (95% CI)* | P value |
| Never user | 13301 | 1956031 | 1 (Reference) |  | 11790 | 1751057 | 1 (Reference) |  |
| 2 years of use | 3015 | 108382 | 2.24 (2.14-2.34) | <0.001 | 2869 | 101588 | 1.99 (1.90-2.09) | <0.001 |
| Remaining years of use | 8686 | 448935 | 1.37 (1.44-1.57) | <0.001 | 8254 | 151858 | 1.17 (1.12-1.22) | <0.001 |
| Recent user** | 2036 | 88326 | 1.64 (1.56-1.72) | <0.001 | 1931 | 82433 | 1.40 (1.33-1.48) | <0.001 |
| Previous user | 15207 | 760750 | 1.33 (1.29-1.37) | <0.001 | 14369 | 714961 | 1.14 (1.10-1.18) | <0.001 |
| Restricted follow-up**** | No. of events | Person-Years | Unadjusted HR (95% CI) | P value | No. of events | Person-Years | Adjusted HR (95% CI)** | P value |
| Never user | 3020 | 5776889 | 1 (Reference) |  | 2364 | 4789203 | 1 (Reference) |  |
| 2 years of use | 520 | 283572 | 1.97 (1.78-2.17) | <0.001 | 486 | 258190 | 1.70 (1.52-1.91) | <0.001 |
| Remaining years of use | 1414 | 853601 | 1.28 (1.19-1.38) | <0.001 | 1300 | 783162 | 0.92 (0.84-1.00) | 0.05 |
| Recent user** | 321 | 133939 | 1.55 (1.37-1.74) | <0.001 | 299 | 120870 | 1.21 (1.06-1.38) | 0.005 |
| Previous user | 2211 | 614314 | 1.32 (1.23-1.41) | <0.001 | 1970 | 540754 | 0.96 (0.88-1.04) | 0.30 |

*Adjusted for principal components 1-5 (in the analysis including all women with different ethnic backgrounds), year of birth, Townsend deprivation index, number of live births, number of stillbirths, polycystic ovary syndrome, age at menarche, age at sexual debut, and family history of depression (excluding the number of live births and stillbirths when the follow-up was restricted). **Cessation of oral contraceptives within two years. ***Assessment of depression from mental health questionnaire. ****Parous women were followed up until one year before delivery.

Table S8. Time-dependent effects of oral contraceptive use among white participants

| Depression | No. of events | Person-Years | Unadjusted HR (95% CI) | P value | No. of events | Person-Years | Adjusted HR (95% CI)* | P value |
| --- | --- | --- | --- | --- | --- | --- | --- | --- |
| Never user | 6154 | 6454498 | 1 (Reference) |  | 4827 | 5376971 | 1 (Reference) |  |
| 2 years of use | 671 | 340767 | 1.88 (1.71-2.05) | <0.001 | 616 | 308507 | 1.64 (1.49-1.81) | <0.001 |
| Remaining years of use | 2935 | 1567764 | 1.31 (1.25-1.37) | <0.001 | 2732 | 308507 | 0.90 (0.86-0.95) | <0.001 |
| Recent user** | 799 | 323877 | 1.55 (1.43-1.67) | <0.001 | 746 | 292394 | 1.13 (1.04-1.23) | 0.003 |
| Previous user | 12360 | 2579380 | 1.50 (1.45-1.55) | <0.001 | 11223 | 2320789 | 1.05 (1.00-1.09) | 0.03 |
| Mental health questionnaire | No. of events | Person-Years | Unadjusted HR (95% CI) | P value | No. of events | Person-Years | Adjusted HR (95% CI)* | P value |
| Never user | 12814 | 1890834 | 1 (Reference) | <0.001 | 11386 | 1698999 | 1 (Reference) |  |
| 2 years of use | 2939 | 105596 | 2.23 (2.14-2.33) | <0.001 | 2801 | 99122 | 2.02 (1.92-2.12) | <0.001 |
| Remaining years of use | 8485 | 438004 | 1.37 (1.33-1.41) | <0.001 | 8071 | 412473 | 1.13 (1.09-2.17) | <0.001 |
| Recent user** | 1984 | 85974 | 1.63 (1.55-1.72) | <0.001 | 1887 | 80367 | 1.40 (1.33-1.48) | <0.001 |
| Previous user | 14869 | 752627 | 1.33 (1.28-1.37) | <0.001 | 14054 | 699177 | 1.13 (1.09-1.17) | <0.001 |
| Restricted follow-up**** | No. of events | Person-Years | Unadjusted HR (95% CI) | P value | No. of events | Person-Years | Adjusted HR (95% CI)** | P value |
| Never user | 2871 | 5424952 | 1 (Reference) |  | 2264 | 4560436 | 1 (Reference) |  |
| 2 years of use | 508 | 273330 | 1.93 (1.75-2.14) | <0.001 | 474 | 249700 | 1.70 (1.51-1.91) | <0.001 |
| Remaining years of use | 1364 | 826812 | 1.24 (1.15-1.34) | <0.001 | 1258 | 760293 | 0.93 (0.85-1.02) | 0.16 |
| Recent user** | 313 | 128826 | 1.53 (1.35-1.73) | <0.001 | 291 | 116538 | 1.23 (1.07-1.40) | 0.003 |
| Previous user | 2152 | 591416 | 1.31 (1.22-1.40) | <0.001 | 1923 | 522274 | 0.97 (0.90-1.06) | 0.49 |

*Adjusted for year of birth, Townsend deprivation index, number of live births, number of stillbirths, polycystic ovary syndrome, age at menarche, age at sexual debut, and family history of depression (excluding the number of live births and stillbirths when the follow-up was restricted). **Cessation of oral contraceptives within two years. ***Assessment of depression from mental health questionnaire. ****Parous women were followed up until one year before delivery.

Table S9. Time-dependent effects of oral contraceptive use excluding women with a medical indication for oral contraceptive use and other psychiatric disorders

| Depression | No. of events | Person-Years | Unadjusted HR (95% CI) | P value | No. of events | Person-Years | Adjusted HR (95% CI)* | P value |
| --- | --- | --- | --- | --- | --- | --- | --- | --- |
| Never user | 4449 | 5818201 | 1 (Reference) |  | 3526 | 4834863 | 1 (Reference) |  |
| 2 years of use | 517 | 312104 | 1.97 (1.79-2.17) | <0.001 | 476 | 280926 | 1.70 (1.53-1.90) | <0.001 |
| Remaining years of use | 2265 | 1431700 | 1.36 (1.29-1.44) | <0.001 | 2107 | 1304848 | 0.93 (0.88-1.00) | 0.03 |
| Recent user** | 610 | 297809 | 1.57 (1.44-1.72) | <0.001 | 568 | 267267 | 1.14 (1.04-1.25) | 0.006 |
| Previous user | 9643 | 2374944 | 1.49 (1.43-1.55) | <0.001 | 8708 | 2125766 | 1.02 (0.98-1.07) | 0.36 |

*Adjusted for principal components 1-5, year of birth, Townsend deprivation index, number of live births, number of still births, age at menarche, age at sexual debut and family history of depression. **Cessation of oral contraceptives within two years. The relevant ICD10 codes for exclusion include: E28 (ovarian dysfunction), N94 (dysmenorrhea), N80 (endometriosis), F31 (bipolar affective disorder), F20 (schizophrenia), F42 (obsessive-compulsive disorder), F43 (posttraumatic stress disorder).

Table S10. Time-dependent effects of oral contraceptive use in adolescence and adults separately

| Time-dependent effects of oral contraceptive use in adolescents | | | | | | | | |
| --- | --- | --- | --- | --- | --- | --- | --- | --- |
| Depression | No. of events | Person-Years | Unadjusted HR (95% CI) | P value | No. of events | Person-Years | Adjusted HR (95% CI)* | P value |
| Never user | 4592 | 3759547 | 1 (Reference) |  | 3516 | 3020860 | 1 (Reference) |  |
| 2 years of use | 297 | 146286 | 2.58 (2.23-3.00) | <0.001 | 283 | 136366 | 1.91 (1.61-2.26) | <0.001 |
| Remaining years of use | 1976 | 855708 | 1.91 (1.80-2.03) | <0.001 | 1863 | 797094 | 1.04 (0.97-1.12) | 0.28 |
| Recent user** | 418 | 131388 | 2.26 (2.03-2.50) | <0.001 | 398 | 122559 | 1.22 (1.10-1.37) | <0.001 |
| Previous user | 5590 | 1012650 | 2.22 (2.13-2.32) | <0.001 | 5181 | 941717 | 1.18 (1.12-1.25) | <0.001 |
| Time-dependent effects of oral contraceptive use in adults | | | | | | | | |
| Depression | No. of events | Person-Years | Unadjusted HR (95% CI) | P value | No. of events | Person-Years | Adjusted HR (95% CI)* | P value |
| Never user | 5349 | 5216735 | 1 (Reference) |  | 4191 | 4209582 | 1 (Reference) |  |
| 2 years of use | 398 | 210767 | 1.77 (1.58-1.98) | <0.001 | 352 | 184750 | 1.64 (1.45-1.86) | <0.001 |
| Remaining years of use | 1132 | 794103 | 0.97 (0.91-1.04) | 0.42 | 1003 | 701362 | 0.79 (0.73-0.85) | <0.001 |
| Recent user** | 416 | 206840 | 1.31 (1.19-1.46) | <0.001 | 376 | 181165 | 1.14 (1.02-1.27) | 0.03 |
| Previous user | 7128 | 1670164 | 1.20 (1.15-1.25) | <0.001 | 6326 | 1460520 | 1.00 (0.95-1.04) | 0.71 |

*Adjusted for principal components 1-5, year of birth, Townsend deprivation index, number of live births, number of still births, polycystic ovary syndrome, age at menarche, age at sexual debut and family history of depression. **Cessation of oral contraceptives within two years.Table S11. Ever users only analysis

| Depression | No. of events | Person-Years | Unadjusted HR (95% CI) | P value | No. of events | Person-Years | Adjusted HR (95% CI)* | P value |
| --- | --- | --- | --- | --- | --- | --- | --- | --- |
| Before initiating OC | 1467 | 4424773 | 1 (Reference) |  | 1334 | 3906257 | 1 (Reference) |  |
| 2 years of use | 771 | 387715 | 2.35 (2.12-2.59) | <0.001 | 700 | 345650 | 1.93 (1.71-2.17) | <0.001 |
|  |  |  |  |  |  |  |  |  |
| Depression** | No. of events | Person-Years | Unadjusted HR (95% CI) | P value | No. of events | Person-Years | Adjusted HR (95% CI)*** | P value |
| Before initiating | 1428 | 4225143 | 1 (Reference) |  | 1298 | 3753456 | 1 (Reference) |  |
| 2 years of use | 747 | 371418 | 2.29 (2.07-2.54) | <0.001 | 681 | 332956 | 1.92 (1.70-2.17) | <0.001 |

2 years of use = the hazard ratio within 2 years after initiation compared to before initiating OC use. *Adjusted for principal components 1-5, year of birth, Townsend deprivation index, number of live births, number of still births, polycystic ovary syndrome, age at menarche, age at sexual debut and family history of depression. **Analysis on women in UK Biobank that self-identified as white. ***Adjusted for year of birth, Townsend deprivation index, number of live births, number of still births, polycystic ovary syndrome, age at menarche, age at sexual debut, and family history of depression.

Table S12. ICE FALCON sensitivity analysis

|  | Coefficient | Model 1 | | | Model 2 | | | Model 3 | | | Change | |
| --- | --- | --- | --- | --- | --- | --- | --- | --- | --- | --- | --- | --- |
| OC use as predictor |  | *β* (SE) | 95% CI | P value | *β* (SE) | 95% CI | P value | *β* (SE) | 95% CI | P value | *β* | P value |
|  | *β* self | 0.51 (0.14) | 0.23-0.80 | <0.001 |  |  |  | 0.48 (0.15) | 0.19-0.76 | 0.001 | -0.036 | 0.16 |
|  | *β* co-sibling |  |  |  | 0.29 (0.15) | 0.001-0.58 | 0.05 | 0.19 (0.15) | -0.10-0.48 | 0.21 | -0.10 | <0.001 |
